# Humoral and cellular immune response to *Plasmodium vivax* VIR recombinant and synthetic antigens in individuals naturally exposed to *P. vivax* in the Republic of Korea

**DOI:** 10.1186/s12936-021-03810-2

**Published:** 2021-06-28

**Authors:** Sanghyun Lee, Young-Ki Choi, Youn-Kyoung Goo

**Affiliations:** 1 Division of Bio Bigdata, Department of Precision Medicine, Korea National Institute of Health, Korea Disease Control and Prevention Agency, Cheongju, Chungbuk 28159 Republic of Korea; 2 Department of Microbiology, College of Medicine and Medical Research Institute, Chungbuk National University, Cheongju, 28644 Republic of Korea; 3 Department of Parasitology and Tropical Medicine, School of Medicine, Kyungpook National University, Daegu, 41944 Republic of Korea

**Keywords:** *Plasmodium vivax*, Variant interspersed repeats protein, Cellular immune response, Humoral immune response

## Abstract

**Background:**

*Plasmodium vivax* proteins with variant interspersed repeats (VIR) are the key proteins used by the parasite to escape from the host immune system through the creation of antigenic variations. However, few studies have been done to elucidate their role as targets of immunity. Thus, this study evaluated the naturally-acquired immune response against VIR proteins in vivax malaria-infected individuals in the Republic of Korea (ROK).

**Methods:**

Seven recombinant VIR proteins and two synthetic peptides previously studied in other countries that elicited a robust immune response were used to investigate the antibody and cellular immune response in 681 *P. vivax*-infected people in ROK. The expression of IgM, IgG, and IgG subclasses against each VIR antigen or against PvMSP1-19 was analysed by ELISA. PvMSP1-19, known as a promising vaccine candidate of *P. vivax*, was used as the positive control for immune response assessment. Furthermore, the cellular immune response to VIR antigens was evaluated by in vitro proliferative assay, cellular activation assay, and cytokine detection in mononuclear cells of the *P. vivax*-infected population.

**Results:**

IgM or IgG were detected in 52.4% of the population. Among all the VIR antigens, VIR25 elicited the highest humoral immune response in the whole population with IgG and IgM prevalence of 27.8% and 29.2%, respectively, while PvMSP1-19 elicited even higher prevalence (92%) of IgG in the population. As for the cellular immune response, VIR-C2, PvLP2, and PvMSP1-19 induced high cell activation and secretion of IL-2, IL-6, IL-10, and G-CSF in mononuclear cells from the *P. vivax*-infected population, comparable with results from PvMSP1-19. However, no significant proliferation response to these antigens was observed between the malaria-infected and healthy groups.

**Conclusion:**

Moderate natural acquisition of antibody and cellular responses in *P. vivax*-infected Korean malaria patients presented here are similar to that in other countries. It is interesting that the immune response to VIR antigens is conserved among malaria parasites in different countries, considering that VIR genes are highly polymorphic. This thus warrants further studies to elucidate molecular mechanisms by which human elicit immune response to the malaria parasite VIR antigens.

**Supplementary Information:**

The online version contains supplementary material available at 10.1186/s12936-021-03810-2.

## Background

*Plasmodium vivax* malaria remains an important problem in central and southeast Asia and South America, with more than 2 billion people at risk of infection [1, 2]. Endemic regions in the Republic of Korea (ROK) are mainly found around the demilitarized zone at the border with the Democratic People’s Republic of Korea. The area had been declared malaria-free by ROK and the World Health Organization in the 1970s, but malaria cases re-emerged in the 1990s [3]. While efforts have been taken to treat and prevent this disease, more than 500 cases occur every year up to this point. Moreover, the introduction of chloroquine-resistant *P. vivax* has been reported in ROK [4]. An effective malaria vaccine capable of inducing a robust and long-lasting protection in naturally exposed individuals should be sought after. Studies evaluating immune responses against different *P. vivax* antigens will aid in the process of vaccine development.

Variant surface antigens (VSAs) of many *Plasmodium* spp. are the key proteins used by the parasites to escape from the host immune system [5]. *Plasmodium falciparum* erythrocyte membrane protein 1 (PfEMP1) and variant interspersed repeat (VIR) proteins encoded by multigene families located on telomeric and subtelomeric regions of the parasite’s chromosomes have been recognized in *P. falciparum* and *P. vivax*, respectively [6]. In *P. vivax*, 346 *vir* genes are divided into 12 subfamilies, named A to L [7, 8]. VIR proteins are exported to the infected erythrocyte’s surface for host immune system evasion, and in reticulocytes, they partially induce the infected cell’s adherence to the endothelial cell receptors [9–11]. VIR antigens are also reported to induce the natural acquisition of antibody-producing and T cell memory responses to *P. vivax* parasites important in *P. vivax* exposed and pregnant populations [12, 13]. In ROK, previously, four VIR genes were studied to understand their genetic diversity, in which the genes showed moderate diversity levels [14]. However, immune responses induced by VIR proteins in *P. vivax*-infected or exposed groups have not been studied. Therefore, the current study was performed to investigate antibody prevalence and host immune responses to VIR proteins in *P. vivax*-infected populations of ROK.

## Methods

### Sample collection

The samples were collected at hospitals and health centers near the demilitarized zone throughout the northern region of the Republic of Korea, where *P. vivax* is endemic in the summer season (June to August). The admission and clinical management of the patients were undertaken independently after blood collection and diagnosis. A total of 681 venous blood samples were collected between 2011 to 2019. In some samples, the *P. vivax* parasite was not observed using microscopy due to low parasitaemia, even though the rapid diagnostic test and nested-polymerase chain reaction (PCR) were positive [15]. The blood samples were centrifuged at 1500*g* for 15 min to separate erythrocytes and serum for further studies. Among the 681 patients, more than 15 ml of venous blood samples were collected aseptically in heparinized tubes from 25 patients (10, 10, and 5 samples from 2017, 2018, and 2019, respectively) for the proliferation and cellular activation assay. Samples were also obtained from 30 healthy volunteers who resided in non-endemic areas (southern parts) of the ROK and had not travelled to *P. vivax* endemic areas. The plasma samples were transported on ice to a laboratory in the Department of Parasitology and Tropical Medicine at Kyungpook National University and were stored at − 70 °C until use.

### Recombinant VIR proteins, MSP1-19 protein and two synthetic peptides

The seven VIR (VIR-A4, VIR-B10, VIR-C1, VIR-C2, VIR25, VIR14 and VIR2) proteins and two synthetic peptides previously used to probe for an immune response against *P. vivax* were prepared for this study [12, 13]. Recombinant VIR proteins were generated as glutathione S-transferase fusion proteins per protocol described previously with modifications [5]. Briefly, PCR products were inserted in the pGEX-4T-3 vector (GE Healthcare, UK), and the sequences were confirmed by standard double-stranded DNA sequencing before expression. Recombinant *Escherichia coli* BL21 (DE3) was grown at 37 °C and 250 rpm in multiple flasks containing 500 ml of LB-ampicillin. When the preparation reached an OD_600_ = 0.4–0.6, isopropyl-β-d thiogalactopyranoside (IPTG, Invitrogen, New Zealand) was added to a final concentration of 2 mM. Cultures were incubated at 18 °C and 250 rpm for 16 h, and bacterial pellets were obtained by centrifugation and resuspended in appropriate volume of sonication buffer [10 mM Tris–HCl pH 8.0, 150 mM NaCl, 1 mM EDTA, 100 μg/ml lysozyme, and 1% Triton X-100]. Bacteria were lysed on ice with the aid of a sonicator. Bacterial lysates were centrifuged at 12,000*g* for 10 min at 4 °C. Recombinant proteins were purified from the supernatant of the bacterial lysates using Glutathione Sepharose 4B beads (Amersham Biosciences, Sweden) and concentrated using Amicon Ultra-15 Centrifugal Filter Units (Merck, Germany). The recombinant GST-fused MSP1-19 representing the 19 kDa C-terminal region of the MSP-1 was expressed in *E. coli* and purified as previously described [16]. Their purity was determined by SDS-PAGE (Additional file 1: Fig. S1). As a control, GST was produced alone. *P. vivax* long synthetic peptides (PvLP) representing the conserved central core (PvLP1) and C-terminal (PvLP2) VIR motifs were designed and synthesized as previously reported [10].

### Quantification of IgG antibodies

A standard ELISA assay was performed as described previously [12, 13]. Briefly, 96-well plates (Nunc, Denmark) were coated overnight at 4 °C with 100 ng/well of each protein in a coating buffer (0.05 M carbonate buffer, pH 9.6). The plates were then blocked with a 3% (w/v) skim milk solution for 1 h at 37 °C. After washing, the plates were incubated at 37 °C for 1 h with serum samples diluted to 1:200. The bound antibody was detected by treatment with HRP-conjugated (BETHYL, USA) anti-human IgG (1:3000), IgG1 (1:1000), IgG2 (1:1000), IgG3 (1:1000), IgG4 (1:500) or IgM (1:10,000) and ABTS [2,2ʹ-azinobis (3-ethylbenzthiazoline sulfonic acid)] (Sigma). The colour was developed at room temperature. Optical density (OD) was measured using an MTP-500 microplate reader (Corona Electric, Japan) at 492 nm. Cut-off points were calculated by the mean OD value plus threefold standard deviations of OD values of sera from 30 healthy volunteers.

### Peripheral blood mononuclear cells (PBMC) proliferation assay

The proliferation assays were performed as previously described [17]. After dilution with the equal volumes of PBS, PBMC were isolated using Ficoll-Paque (Amersham Biosciences, Sweden) and centrifugation. The PBMC at the interface were collected, washed three times in PBS, and resuspended in complete media. Viable PBMC counts were made under a phase-contrast microscope using the trypan blue dye exclusion test. The complete media was RPMI 1640 medium (Invitrogen, New Zealand) supplemented with 10% normal human serum, 2 mM l-glutamine, 10 mM HEPES, 0.2% sodium bicarbonate, and 100 U/ml penicillin and streptomycin. A total of 2.0 × 10^5^ cells in 200 μl of culture media were added to each well of a flat bottom 96-well plate (Corning Co., NY). Each recombinant VIR protein, synthetic peptides, MSP1-19, or GST, were added in 20 μl amounts. The final concentration of the recombinant proteins or GST was 20 μg/ml. The assay was performed in triplicate cultures, including an extra well containing only culture medium for each assay. Concanavalin A (Sigma, USA) was used as a positive control in all the experiments. Cultures were incubated in a humid environment, at 37 °C, in a 5% CO_2_ atmosphere for 2 days. After 96 h, the cultures were harvested and counted in Cell Counting Kit-8 (Dojindo Laboratories, Japan) reagent.

### Determination of cellular activation

Cellular activation was determined using monoclonal antibodies against CD4, CD8, and CD25. Briefly, the diluted antibody was added to 2 × 10^5^ stimulated or non-stimulated mononuclear cells in 50 μl PBS containing 2% fetal calf serum and 0.1% sodium azide, and then incubated for 20 min at 4 °C. After washing twice with PBS, the cells were resuspended in PBS with sodium azide, fixed with a 2% paraformaldehyde solution, and then maintained in the dark until analysed by flow cytometry.

### Cytokine detection assay

IL-2, IL-6, IL-10, IL-12, IFN-γ, TNF, IP-10, and G-CSF in plasma and supernatant of PBMC culture samples were measured using commercial ELISA kits (Thermo Fisher Scientific, USA).

### Statistical analysis

Data was analysed by using SPSS software package version 17.0. The differences between the proportions of responders were analysed using the Chi-square test. The significance level was set at *P* < 0.05.

## Results

### Characteristics of study population

A total of 681 peripheral blood samples collected from *P. vivax*-infected patients between 2011 and 2019 (253, 199, 107, 27, 21, 23, 21, 25, and 16, in chronological order). According to regions, the samples were obtained from Gang-wha (n = 193), Paju (n = 253), Kimpo (n = 122), and Yeonchen (n = 113). The mean age of this study group was 30.3 ± 11.2 years old, and 78.1% of the subjects were male. Thirty healthy controls were collected from Daegu (n = 30). The mean age of this control group was 25.1 ± 3.2 years old, and 30% of the volunteers were male.

### Antibody responses to VIR proteins and synthetic peptides

Serum samples collected from 681 individuals living in the northern parts of ROK and infected by *P. vivax* were tested by ELISA (using seven GST-fusion VIR proteins and two synthetic peptides as coating antigens) for the presence of IgM, IgG, and IgG subclasses antibodies. The results showed that the frequency of individuals exhibiting positive antibody response to a recombinant VIR ranged from 6.6% (45 out of 681 individuals, for VIR2) to 27.8% (for VIR25) (Table 1). Among VIR antigens, VIR25 showed the highest significant response in the form of IgG and IgM in infected individuals (*P* < 0.05, Chi-Square test), followed by VIR-C subfamily members (VIR-C1 and VIR-C2) (Table 1). The positive control, recombinant MSP1-19, elicited IgG antibodies in 621 individuals, resulting in the frequency of 91.2% for humoral immune response (Table 1).

**Table 1:** Prevalence of the antibody response in 681 individuals infected with *Plasmodium vivax*

Recombinant protein or synthetic peptides	Prevalence of positive sera (%)	
	IgG (n = 681)	IgM (n = 681)
VIR-A4	91 (13.4)	123 (18.1)
VIR-B10	103 (15.1)	116 (17.0)
VIR-C1	123 (18.1)	113 (16.6)
VIR-C2	157 (23.1)	146 (21.4)
VIR25	189 (27.8)	199 (29.2)
VIR14	82 (12.0)	56 (8.2)
VIR2	45 (6.6)	43 (6.3)
PvLP1	71 (10.4)	58 (8.5)
PvLP2	79 (11.6)	89 (13.1)
MSP1-19	621 (91.2)	ND

Subsequently, the IgG subclasses were determined by subclass-specific ELISA using the recombinant proteins and peptides with samples that showed IgG positive. There is significantly higher prevalence of IgG1 against VIR-C1, VIR-C2, VIR25 and PvLP1 than IgG1 against other VIR antigens, and IgG1 is also the major subclass of IgG that targets VIR-C1, VIR-C2, VIR25 and PvLP1. Moreover, VIR-A4 turns out to be the only VIR antigen that elicited the prevalence of over 20% for all four subclasses of IgG (i.e., IgG1, IgG2, IgG3 and IgG4) (Table 2).

**Table 2:** Prevalence of IgG subclass responses to VIR antigens

IgG subclass	Percentage of positive sera (%)								
	VIR-A4 (n = 91)	VIR-B10 (n = 103)	VIR-C1 (n = 123)	VIR-C2 (n = 157)	VIR25 (n = 189)	VIR14 (n = 82)	VIR2 (n = 45)	PvLP1 (n = 71)	PvLP2 (n = 79)
IgG1	22.0	9.7	45.5	37.6	32.8	14.6	17.8	29.6	15.2
IgG2	23.1	4.9	13.0	13.4	11.1	23.2	22.2	14.1	12.7
IgG3	26.4	8.7	12.2	10.2	8.5	18.3	24.4	11.3	19.0
IgG4	22.0	5.8	17.1	16.6	13.8	14.6	24.4	15.5	15.2

### Proliferative and cellular responses of VIR proteins and synthetic peptide

**Fig. 1:**
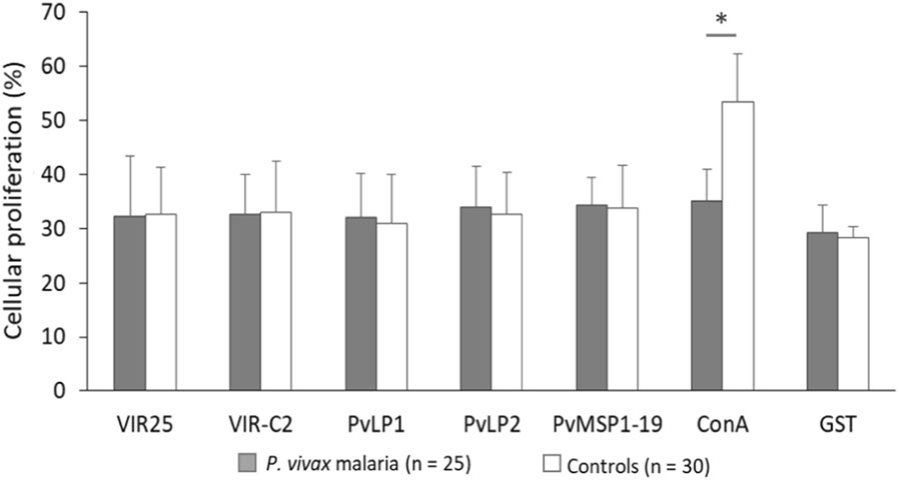
PBMC proliferation response to recombinant proteins and synthetic peptides of VIR after 96 h culture. PBMC from *P. vivax* malaria patients and control individuals in the absence or presence of plasmodial antigens or ConA (*P < 0.05 for *P. vivax*-infected patients versus control individuals)

PBMC collected from 25 *P. vivax* malaria patients were tested for proliferation in the presence of any of the following VIR proteins or synthetic peptides: VIR-C2, VIR25, PvLP1 and PvLP2. The proteins and peptides were selected based on high antibody responses (IgG and IgM) in the ELISA results above. In parallel, GST or MSP1-19 were also used as controls. GST, MSP1-19, and ConA (a known inducer of PBMC proliferation) were used as controls for the proliferation test. Except for ConA, neither MSP1-19 nor any VIR protein or synthetic peptide elicited PBMC proliferation (Fig. 1).

### Cellular responses to VIR proteins, PvPL1, and PvLP2

**Fig. 2:**
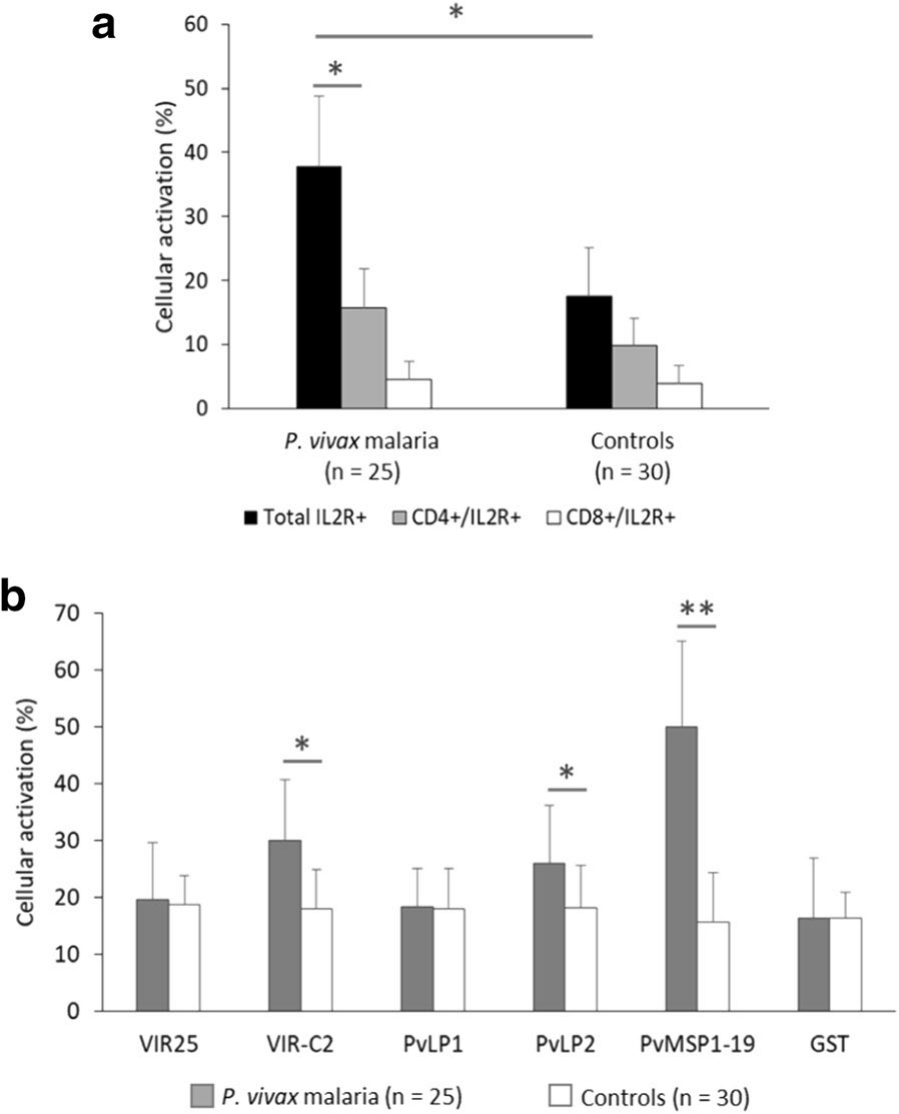
Analysis of cellular activation by the expression of IL2R after 96 h culture in absence (**A**) and presence of recombinant proteins or synthetic peptides of VIR (**B**) by cytometry. PBMC from malaria patients (n = 25) and control individuals (n = 15) (*P < 0.05 for *P. vivax*-infected patients versus control individuals; **P < 0.001 for *P. vivax*-infected patients versus control individuals)

The phenotype of PBMC from *P. vivax*-infected individuals revealed that the percentage of CD4^+^ T cells was higher than CD8^+^ cells, which was the same in the control group (data not shown). Then, cellular activation was determined by the expression of the interleukin-2 receptor (IL-2R), using an anti-CD25 monoclonal antibody. In the ex-vivo analysis, PBMC from *P. vivax*-infected individuals presented higher levels of cellular activity than healthy individuals (*P* < 0.05) (Fig. 2A). In individuals infected with *P. vivax*, CD4^+^ T cells were more activated than the CD8^+^ cells (*P* < 0.05) (Fig. 2A). Significantly higher levels of cellular activation were observed in *P. vivax*-infected groups stimulated with VIR-C2 (*P* < 0.05), PvLP2 (*P* < 0.05), or PvMSP1-19 (*P* < 0.001) than in controls (Fig. 2B). However, the groups tested with VIR25 and PvLP1 showed similar cellular activation between *P. vivax*-infected and control individuals.

Subsequently, the concentrations of cytokines, chemokines, and growth factors secreted by PBMC of the study participants in the presence of VIR proteins, PvLP1, PvLP2, PvMSP1-19, GST or culture medium were assessed. As shown in Table 3, PBMC from *P. vivax* malaria patients secrete significantly higher levels of IL-2, IL-6, IL-10, TNF and G-CSF than PBMC from healthy donors when they are stimulated by VIR-C2, PvLP2 or PvMSP1-19, but not by any others. Additionally, in the presence of VIR-C2, PBMC from *P. vivax* malaria patients secrete significantly higher levels of IL-12 than PBMC from healthy donors.

**Table 3:** Median concentration (pg/ml) values for each cytokine and growth factor in plasma of *P. vivax*-infected and uninfected groups

	IL-2	IL-6	IL-10	IL-12	IFN- γ	TNF	IP-10	G-CSF
VIR25								
* P. vivax*	10	27	34	42	14	62	10	98
Healthy control	13	19	31	46	11	56	12	102
* P* values	0.225	0.092	0.103	0.312	0.098	0.236	0.168	0.263
VIR-C2								
* P. vivax*	89**	41*	102*	74*	13	112*	12	195*
Healthy control	11	20	33	42	12	64	9	112
* P* values	< 0.001	0.029	0.018	0.035	0.156	0.031	0.264	0.039
PvLP1								
* P. vivax*	7	29	52	40	11	72	13	124
Healthy control	10	12	39	41	12	69	10	96
* P* values	0.265	0.314	0.247	0.184	0.206	0.105	0.241	0.198
PvLP2								
* P. vivax*	32*	72*	132**	61	13	103*	13	159*
Healthy control	12	19	49	49	11	64	15	95
* P* values	0.045	0.013	< 0.001	0.235	0.153	0.031	0.128	0.015
PvMSP1-19								
* P. vivax*	125^**^	95**	89**	58	15	120*	49	201*
Healthy control	16	14	39	46	13	62	45	106
* P* values	< 0.001	< 0.001	< 0.001	0.391	0.153	0.013	0.364	0.041
GST								
* P. vivax*	10	25	38	51	14	67	10	116
Healthy control	12	16	31	41	12	62	11	102
* P* values	0.125	0.312	0.186	0.216	0.234	0.163	0.113	0.265
Medium								
* P. vivax*	10	39	35	50	13	70	10	121
Healthy control	16	15	32	45	15	68	9	110
* P* values	0.315	0.132	0.327	0.192	0.264	0.162	0.298	0.225

n = 25 for *P. vivax*-infected groups; n = 30 for healthy control

^*^P < 0.05 for *P. vivax*-infected patients versus control individuals

^**^P < 0.001 for *P. vivax*-infected patients versus control individuals

## Discussion

This study describes the immune response of individuals infected with *P. vivax* to recombinant proteins and synthetic peptides representing *P. vivax* variant antigens (VIR). This is the first report showing antigenic properties and immune characteristics of VIR antigens in ROK after the genetic diversity of *vir* genes was evaluated in ROK [14]. The seven VIR proteins and two synthetic peptides used in the current study were selected based on their results of the high prevalence and broad distribution in previous studies [12, 13].

First, the frequency of individual serum samples containing IgM or IgG antibodies to each of the VIR antigens was determined. The results showed that a proportion of infected samples recognized the recombinant proteins or synthetic peptides was various depending on the tested antigens (IgM, from 6.3 to 29.2%; IgG, from 6.6 to 27.8%). This detection rate was higher than those in a previous study done in Brazil (IgM, from 6.0 to 18.1%, IgG, from 2.0 to 17.5%) [12]. Since there are high genetic diversity and high gene copy number per haploid genome of the VIR proteins in natural parasite populations, the results indicate high immunogenicity of VIR proteins during natural human infections [5, 9, 18]. Among VIR proteins, VIR-C2 and VIR25 elicited high prevalence of IgG and IgM in the current study, similar to findings from other groups [12, 13], indicating that the B cell epitope might be sufficiently conserved worldwide despite the high sequence diversity in VIR proteins. VIR-C2 is a member of subfamily C. It was reported that the gene encoding VIR4, another subfamily C member, is conserved in the *P. vivax* parasites in ROK, India, and Myanmar [14, 19, 20]. In contrast to the high prevalence of IgG and IgM to VIR proteins, a response to synthetic peptides was not detected.

Several studies with *P. falciparum* and *P. vivax* have demonstrated a positive correlation between IgG response and patient age in endemic malaria regions caused by the agglutinating antibodies produced [21–23]. Furthermore, the immune response seems to have cross-reactivity with diverse isolates [24, 25]. In current study, the establishment of association relationship between antibody levels specific for VIR proteins or synthetic peptides and patient age was failed, owing to the limitation of age structure of the study participants. Specifically, 426 out of 681 participants were soldiers residing in military bases with the age of 20–30 years old. In contrast, only 23 participants (3.4%) were over 60 for age.

Interestingly, IgG1 was found to be the dominant subclass of IgG that displayed high prevalence of total IgG response against VIR-C1, VIR-C2, VIR25, and PvLP1. Moreover, high response rates of IgG3 and IgG4 (both 24.4%) to VIR2 were observed in the current study. The function of IgG subclass in immune strategy in malaria, such as the opsonization capacity of IgG1 and IgG3, was well documented [26]. Moreover, IgG1 and IgG3 are the dominant subclass involved in the VSA-specific IgG response in adults and children, respectively [27]. Since the samples were collected from patients over 16 years old, age-dependent responses in the IgG subclasses were not evaluated.

Low proliferative responses in the presence of MSP1-19 of *P. falciparum* and *P. vivax* have already been demonstrated [12, 28]. Furthermore, the current results also showed a low proliferative response against variant proteins and peptides and PvMSP1-19 after 96 h in culture. This low proliferative response may occur because most activated cells undergo activation-induced cell death in T lymphocytes that play important roles in peripheral deletion events involved in tolerance and homeostasis by apoptosis of T cell receptor activated cells [29].

It is known that *P. vivax* parasites induce a specific immune response by stimulating the release of cytokines, and this may have an important function in activating the host’s immune cells to react to the parasite [30]. In this study, PBMCs exposed to *P. vivax* and re-stimulated with VIR-C2 and PvLP2 showed higher secretion of IL-2, IL-6, IL-10, and TNF, which was the same pattern in PvMSP1-19. The significant IL-2 cytokine response may assist IL-4/IL-5/IL-13- or IL-17-producing effector helper cells for cell expansion in acute malaria [31, 32]. Furthermore, highly secreted pro-inflammatory cytokines, IL-6 and TNF, in vivax malaria patients represent T cell differentiation toward Th2 and Th17 may be skewed and trigger the natural acquisition of cellular memory immune responses [33]. IL-10, a key regulatory cytokine, prevents excessive inflammation and seemed to increase in lymphocyte culture for a significant amount of time and was elevated in subjects who had recovered from *P. vivax* infection. The increased level of IL-10 in this study after 96 h in culture with VIR-C2 and PvLP2 indicates that regulatory responses may reside within the memory population, and the re-stimulation may be secondary activation of Th1 cells [34, 35].

There are a few studies regarding immune responses to VIR antigens to be compared to this data. Research shows that VIR proteins have been considered as a vaccine target candidate. Being similar to the previous report, the data produced with VIR antigens was not significantly better than the positive control PvMSP1-19. However, it was found that the immune responses are conserved among different countries (ROK, Brazil, and Australia) despite their high genetic diversity [12–14].

## Conclusion

The results presented here show that VIR antigens, especially VIR-C2 and PvLP2, induce the natural acquisition of antibody and cellular response in *P. vivax*-infected individuals in ROK. In consideration that immune response induced by the VIR antigen is conserved among malaria parasites in different countries and VIR genes are highly polymorphic, further study is warranted to elucidate the molecular mechanism by which immune response to VIR antigens is elicited.

## Supplementary Information

**Additional file 1: Figure S1.** SDS-PAGE analysis of the purified recombinant VIR proteins.

## Data Availability

All data generated or analysed during this study are included in this published article.
