# Supplementary figures and images for: Humoral and cellular immune response to Plasmodium vivax VIR recombinant and synthetic antigens in individuals naturally exposed to P. vivax in the Republic of Korea
Source: Malar J. 2021 Jun 28;20:288. doi: 10.1186/s12936-021-03810-2 (PMC8237554; doi:10.1186/s12936-021-03810-2)

## Slide 1
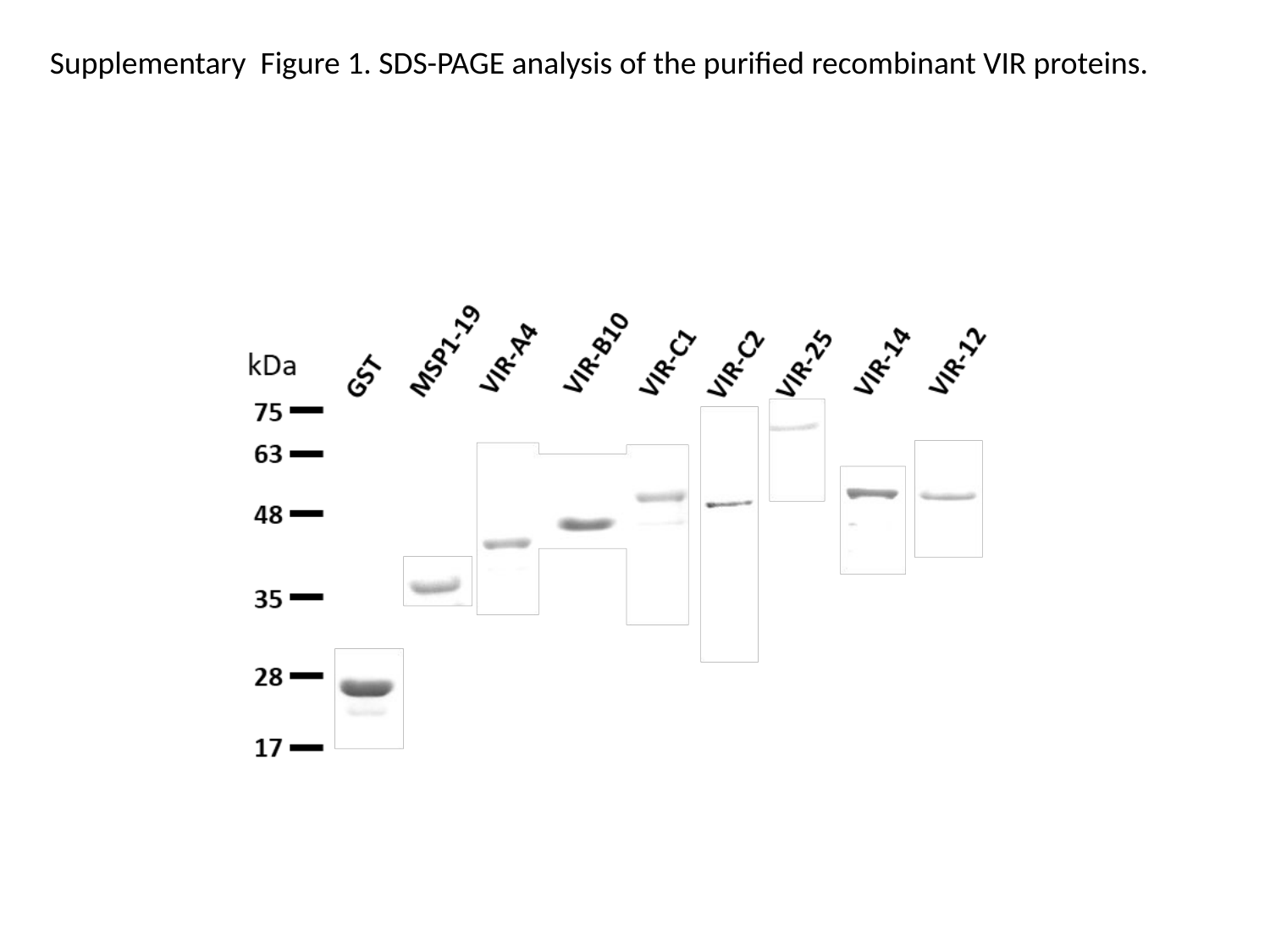

Supplementary Figure 1. SDS-PAGE analysis of the purified recombinant VIR proteins.

Supplement: Supplementary file 1 — Additional file 1: Figure S1. SDS-PAGE analysis of the purified recombinant VIR proteins. [file 12936_2021_3810_MOESM1_ESM.pptx]
